# Handling Sign Language Data: The Impact of Modality

**DOI:** 10.3389/fpsyg.2019.00483

**Published:** 2019-03-12

**Authors:** Josep Quer, Markus Steinbach

**Affiliations:** ^1^ ICREA-Pompeu Fabra University, Barcelona, Spain; ^2^ University of Göttingen, Göttingen, Germany

**Keywords:** sign language, native signers, language modality, language analysis, language documentation, data collection

## Abstract

Natural languages come in two different modalities. The impact of modality on the grammatical structure and linguistic theory has been discussed at great length in the last 20 years. By contrast, the impact of modality on linguistic data elicitation and collection, corpus studies, and experimental (psycholinguistic) studies is still underinvestigated. In this article, we address specific challenges that arise in judgment data elicitation and experimental studies of sign languages. These challenges are related to the socio-linguistic status of the Deaf community and the larger variability across signers within the same community, to the social status of sign languages, to properties of the visual-gestural modality and its interface with gesture, to methodological aspects of handling sign language data, and to specific linguistic features of sign languages. While some of these challenges also pertain to (some varieties of) spoken languages, other challenges are more modality-specific. The special combination of the challenges discussed in this article seems to be a specific facet empirical research on sign languages is faced with. In addition, we discuss the complementarity of theoretical approaches and experimental studies and show how the interaction of both approaches contributes to a better understanding of sign languages in particular and linguistic structures in general.

## Introduction

Sign and spoken languages use two different modalities, the visual-gestural modality of sign languages and the oral-auditory modality of spoken languages. Although the two modalities clearly differ in the production and perception of communicative signals, the underlying linguistic structures seem to be very similar across both modalities (Meier, 2002, 2012; Sandler and Lillo-Martin, 2006).^1^ In addition, psycho- and neurolinguistic studies with non-impaired and impaired deaf signers show that sign languages access the same neural networks involved in auditory speech processing, albeit with some concrete modality-specific features (Poizner et al., 1987; Emmorey, 2002, 2003; Corina and Knapp, 2006; Campbell et al., 2008; Corina and Spotswood, 2012; Dye, 2012; Woll, 2012). Nevertheless, sign languages retain some modality-specific properties that may impact the linguistic structure and the cognitive processes underlying the perception and production of signed communication and that have an influence on the handling of sign language data (cf. van Herreweghe and Vermeerbergen, 2012; Orfanidou et al., 2015). First of all, sign languages employ various articulators such as the hands, the upper part of the body, the head, and the face to express grammatical features simultaneously. Second, sign languages use the geometrical properties of the signing space to realize morphosyntactic, semantic, and pragmatic categories in the three-dimensional signing space (Engberg-Pedersen, 1993; Padden, 1998; Aronoff et al., 2005; Pfau and Steinbach, 2016; Steinbach and Onea, 2016). Third, sign languages grammaticalize and integrate gestural elements, since sign languages and manual as well as non-manual gesture use the same modality. As a consequence, the interface between these two systems is permeable (Liddell and Metzger, 1998; Emmorey, 1999; Liddell, 2003; Pfau and Steinbach, 2011; Grosvald et al., 2012; Goldin-Meadow and Brentari, 2017) and leads to a more prominent presence of iconicity at different grammatical levels (Taub, 2012). By contrast, there is much less transparency between the signals used in auditory communication and their meaning (Schlenker, 2018).

Besides these linguistic differences, sign languages differ from many spoken languages also in various socio-linguistic dimensions (Aronoff et al., 2005). In the next section, we first deal with these dimensions (the data source problem) and discuss consequences of the quite heterogeneous group of sign language users for linguistic studies. In the second part, we turn to the impact of modality on the elicitation and annotation of sign language data. Specific practical and conceptual challenges may arise from the heterogeneity of linguistic informants and subjects, the lack of a writing system, the material properties of the data, and the modality-specific linguistic aspects of sign languages mentioned above. Note that while some of these challenges may also hold true for some varieties of spoken languages (e.g., for spoken languages without a written form used by linguistic minorities in multilingual contexts), other challenges are clearly modality-specific. Since the focus of this article is on sign language data handling, we discuss spoken languages only in passing. It will, however, turn out that the expertise gained in linguistic research on sign languages paves the way for new multimodal investigations of spoken languages.

## The Data Source Problem

Formal linguistic analysis typically relies on evidence provided by native speakers of the language or variety under study. This can involve different types of collected spontaneous or semi-spontaneous productions, elicited utterances, or grammaticality judgments. Despite the unavoidable abstraction across different speakers, it is taken for granted that their competence is similar enough by virtue of having acquired the language natively in a typical, unproblematic fashion.^2^ However, such a simple assumption cannot be made for sign languages because of their highly idiosyncratic sociolinguistic settings and in particular their dominant acquisition patterns (Schembri and Lucas, 2015).

At least for Western societies, it is often taken for granted that only 5–10% of deaf children are born to deaf parents or in an environment where there is adequate sign language input for the child to develop language competence in a natural way (Neidle et al., 2000; Mitchell and Karchmer, 2004). This means that most deaf babies (the remaining 90–95% of deaf children at birth) are not surrounded by a natural language in the visual-gestural modality, which is fully accessible to them, but rather by spoken language. A variety of factors determines the language acquisition path for them: (1) hearing parents can decide to learn and use sign language themselves with the child (a very small percentage, cf. Chen-Pichler and Lillo-Martin, 2018); (2) parents can choose a schooling model that favors interaction and instruction in sign language to different degrees (from deaf schools to bilingual programs embedded in regular schools); (3) parents are often confronted with the choice of giving their child a cochlear implant that will facilitate access to the spoken language signal after regular and intensive training. These elements already make it evident that for most deaf children access to language during the critical period will be uncertain, to say the least, and in any event more incomplete or degraded than in the default case where rich language input is part of the environment. Take for instance the favorable, albeit uncommon, case where parents decide to use sign language with the child and choose for a day care and school that offers a bimodal bilingual approach: even in this favorable case, most adult language models will be non-native (hearing parents, hearing teachers and classroom interpreters that learn sign language as a second language) and some of them will use mixed forms of language (in general, spoken structure imposed on sign), thus providing an input that is strictly speaking qualitatively different from the native one. The obvious consequence of this situation is that the majority of signers in Deaf communities have acquired their sign language under such special circumstances and do not fall under the strict definition of native speakers or signers. To this we must add the fact that regular contact with sign language may happen at different stages in life and it is quite common for deaf children to be initially raised only with spoken language and for them to be exposed to sign language past the first year of life, turning them technically into early or late learners of what normally becomes their main language of communication. In this situation, it is quite often the case that access to spoken language is so limited in early life that late acquisition of a sign language is not L2 learning, but simply delayed L1 learning at an abnormal age (late childhood, adolescence, or adulthood), leading to abnormal neurological mappings of language (Mayberry, 2010; Mayberry and Kluender, 2017; Woll, 2018). Research has confirmed the expectation that such different paths of language acquisition should impact on language competence (Boudreault and Mayberry, 2006; Cormier et al., 2012; Skotara et al., 2012; Hänel-Faulhaber et al., 2014, 2018, unpublished; Lillo-Martin, 2018).

Next to such atypical language acquisition paths, linguistic research must also take into account that most deaf signers have bilingual competence as a result of spoken language acquisition to varying degrees, even if it is the language acquired first chronologically. Nowadays spoken language competence in signers takes two different paths: mostly competence in the written form, as a result of schooling and interaction with the ambient hearing society; competence in the spoken modality because of the spreading of cochlear implants, which typically involves mainstreaming in education and intensive speech therapy. In this picture, postlingual deaf children constitute yet another case, as they will have acquired spoken language for the most part when they lose their hearing, thus being able to rely on full-fledged language acquisition during the first year of life as base for subsequent sign language acquisition.

Among bilingual signers, another group must be taken into account: hearing native signers, most commonly known as bimodal bilinguals (Branchini and Donati, 2016; Emmorey et al., 2016; Lillo-Martin et al., 2016). This population is formed by hearing children of Deaf adults (CODAs) who have been exposed to sign from birth and have acquired it natively while acquiring the ambient spoken language at the same time in the larger family context, at school and in social interaction. CODAs form an idiosyncratic language group that has only received attention quite recently within the study of bilingual competence. In a sense, they represent the unique case of full simultaneous bilingualism in two modalities, given their unproblematic access to the input in both sign and spoken language. They offer a unique window into the bilingual mind that can process and externalize utterances realized in the two channels simultaneously, namely code blends. As for their sign language competence, it has been paralleled to that of heritage speakers, since they will use it only in family or community contexts and will use the ambient spoken language most of the time (unless they become sign language interpreters, of course, or have deaf children or a deaf spouse) (Quadros, 2018).

This cursory description of the factors that impact on the individual competence of sign language users highlights the complexity of trying to characterize language competence across a signing community. It is still common practice – among formal linguists at least – to study sign language structure relying on evidence provided by native signers, even though they constitute a very small minority within signing communities. Their scarcity often involves difficulties in accessing native signers as language consultants that are willing to collaborate and provide data, and in some cases, it cannot even be feasible, as discussed by Costello et al. (2008). The situation might be even more problematic if the usually quoted rates of deaf-of-deaf individuals are in reality lower in countries other than the United States, as argued by Johnston (2006).

Given these limitations, some alternatives have been proposed. One of them consists in working with consultants that get as close as possible to a native signer, as put forth in Mathur and Rathmann (2006): (1) exposure to a sign language by the age of 3; (2) daily contact with a sign language in the Deaf community for longer than 10 years. For linguistic research, they also required (3) capability to make grammaticality judgments with ease. Freel et al. (2011) also establish this age limit of 3 in the acquisition of sign language in order to count someone as native signer. Such accommodations seem desirable in practical terms, but it might be the case that even with these slight departures from strict nativehood, it is still hard to find sign language consultants, given their scarcity in some areas.

An obvious reaction to the difficulty of working with native signers to obtain fresh data would be to resort to existing resources such as grammars and corpora. Unfortunately, such tools do not exist for most sign languages. With a few exceptions, reference works or even partial descriptions of grammar components (phonology, morphology, syntax, etc.) are lacking. An attempt to remedy this situation has been undertaken by developing a detailed guide to sign language grammar writing, the *SignGram Blueprint* (Quer et al., 2017), which will also be implemented as an online grammar writing tool on the platform currently developed by the SIGN-HUB project (H2020: 2016–2020). By the end of this project, the grammars of six languages will be available, and hopefully, this step will set the trend for other sign languages and steadily fill the vast gap that we are currently faced with in terms of background grammatical information for languages in the visual-gestural modality.

Sign language corpora are not available as a default (e.g., there is no reference corpus for American Sign Language (ASL) despite being the longest studied sign language), but different projects in Europe and Australia have addressed this need and developed representative corpora for certain sign languages that gather spontaneous or semi-spontaneous signing on the basis of different tasks or elicitation techniques.^3^ Some of them are already available, while others are currently being built. But even if a corpus is available, one general problem of most corpora is that they lack detailed linguistic annotation, especially at the levels of morphosyntax and (discourse) semantics. Hence, they can be used for linguistic investigations only to a limited extent. A more general problem is the significance of corpora. Although corpus data are useful for the description of grammatical structures and sociolinguistic variation, they are known to be problematic for theoretical analysis, given the limitation that no negative evidence can be obtained (non-appearance in the corpus cannot be equated to ungrammaticality). In the case of sign languages, the individual variation referred to above must be added to the complications of relying on corpus data. The issue can be mitigated, thanks to the use of metadata about the consultants recorded in such a way that one could in principle select only production by signers with a common linguistic profile (e.g., natives). However, the best situation will be one in which data types can be combined, for instance, by collecting corpus data and eliciting grammaticality judgments. Another technique used in sign language research is to discuss data with consultants, whether they have been produced by themselves (and played after an acceptable time lapse) or by others (as with corpus data, for instance).

Having access to native signers as consultants or enough relevant corpus or elicited data, though, is not enough to be able to guarantee that we are researching a particular sign language. As is well known from spoken language research, variation within a linguistic domain needs to be taken into account when defining the object of study. A similar situation arises with sign language data but sometimes with parameters of variation that are unique to the visual-gestural modality.

Geographical variation is certainly also present in sign language communities, but with some idiosyncratic features vis-à-vis spoken languages. Till quite recently, regional variants of sign languages were only indirectly determined by geographical area of use: given the dispersal of deaf individuals within hearing societies, the two poles of emergence and irradiation of signed varieties were mainly: (1) deaf (boarding) schools and (2) deaf clubs. These institutions created contexts where deaf signers formed a critical mass for language use, but crucially also for language acquisition/learning. The impact of educational institutions on variation is clear in many countries, as in the Netherlands, where five regional variants can be identified as a consequence of the existence of five different deaf schools (Schermer, 2012). This kind of variation mainly affects the lexicon (especially in certain lexical domains like numerals, names of weekdays and months, colors, and kinship terms; gender differences can even be traced back to the existence of segregated schooling), phonology, and grammar.

There are very few studies that focus on variation from a formal point of view, but the potential of corpus data analysis from this perspective is clear. One of them deals with the position of wh-elements across regional variants of Italian Sign Language (LIS) (Geraci et al., 2015), and it interestingly concludes that a variable like age (and, linked to that, language awareness) plays a decisive role in the position of wh-elements. With this, we see how sociolinguistic factors such as schooling, language contact and awareness can determine language production (and arguably competence). Another interesting example of research that targets grammatical phenomena relying on data that reflect variation concerns the syntactic position of the agreement auxiliary pam in German Sign Language (DGS) (Macht and Steinbach, 2018): a rough partition of the DGS domain in north, west, east, and south shows that in the former three the preverbal realization of the auxiliary is clearly predominant (72% up to 85% of the instances), while in the south, it appears before and after the predicate in almost the same percentages. These results clearly point to a different syntactic derivation across areas of the same language domain that need to be further investigated with respect to other structural phenomena.

With this brief overview of the individual and social factors that can determine language competence in signers it becomes evident that data elicitation, grammaticality judgments tasks and experimental studies should be carried out with particular care in order to reach reliable generalizations about a particular sign language.

## Modality and Data Collection

In the previous section, we discussed various individual and social factors that may affect any kind of empirical and experimental data collection, annotation, evaluation, and documentation. Some of them are related to the fact that sign languages are minority languages and that deaf native signers form a unique linguistic minority. Others are related to the specific kind of language acquisition, the influence of the ambient spoken language(s), and the (linguistic) heterogeneity of the Deaf community. Before we turn to modality-specific aspects that may have an impact on data collection, we briefly discuss how these aspects need to be considered in empirical investigations of sign languages (for more details, see Orfanidou et al., 2015).

First of all, working with linguistic minorities requires the strict compliance of the highest standards of ethical principles. This is especially important since most kinds of data collection involve video recording, which means that informants or subjects are always visible and clearly identifiable.^4^ Because of the very specific properties of visual-gestural languages, data cannot easily be made anonymous since each part of the upper part of the body and the face conveys important grammatical and pragmatic information. This brings us to the second aspect: Sign language data are typically video data, that is, sign language linguists always use, collect, annotate, and analyze quite complex visual information. As opposed to many spoken languages, sign languages do not have a written form that can be used for data collection and data storage. Linguistic glosses used in research on sign languages are always only simplified linguistic representations of the multidimensional visual information of a corresponding video documenting the utterance (Frishberg et al., 2012; Crasborn, 2015). We will come back to this issue below. Moreover, effective tools for automatic processing and annotation of sign language video data are not available yet (see Hanke, 2016 and below). Third, a careful collection of metadata is inevitable to specify the significance of a specific set of data collected in an empirical study. The validity of data depends on the kind of informants and subjects involved in the study. A related fourth aspect is that each empirical study should start with a clear definition of the socio-linguistic features of informants and subjects of a study to get optimal and valuable empirical data for the linguistic research question under discussion. This is especially important for studies with smaller groups of informants and subjects. Fifth, empirical studies should always be conducted in a sign language-friendly environment, which includes interaction and instruction in sign language, and use deaf friendly research methods. Ideally, the study is conducted by a deaf researcher. Likewise, the data should be annotated and evaluated by mixed teams including deaf researchers. And last but not least, linguists should be aware of the fact that sign language users are not only a linguistic minority but are in many countries and regions also very small groups with many non-academic members. Therefore, any kind of data collection should respect the specific needs of these groups and include regular activities of transfer of knowledge and dissemination in the local sign language.

The heterogeneity of the Deaf community may also directly affect the results of linguistic studies. In many Western societies, sign languages have been recognized only recently. Therefore, informants and subjects may have grown up in bilingual or strict oralist environments where sign languages have not been taught at school (and have even not been used in the classroom). This situation—which is not modality-specific but typical for many sign languages—can have an influence on the evaluation of linguistic data and grammaticality judgments of signers, especially in tasks where certain information (such as, for instance, linguistic contexts) is provided in a spoken language or where answers to linguistic questions have to be given in a spoken language. Like for other bilinguals, it has been shown that deaf bimodal bilinguals also activate the second language (i.e., the ambient spoken language) while processing the first language (i.e., the native sign language) (Hosemann, 2015). Therefore, the specific language awareness in oral environments and the fact that deaf signers are typically bilingual should be taken into account, as mentioned earlier. In general, using spoken language input for elicitation tasks should be avoided if possible in order to minimize interference (Nishio et al., 2010). This means that instructions and contexts necessary for controlled data elicitation have to be provided in the sign language under investigation (for the importance of controlled data elicitation, see Matthewson, 2004).

Let us now turn to modality-specific aspects of sign languages that are relevant in this context. First of all, unlike spoken languages, sign languages are characterized by a relatively long transition phase between two linguistic units (compared to spoken languages).^5^ One reason for this is that sign languages, unlike spoken languages, make use of relatively massive articulators that execute long movements (Meier, 2002, 2012). Consequently, phonological parameters change much slower than in spoken languages. In addition, phonological parameters can be realized simultaneously, that is, in the transition phase more than one parameter may change at the same time. Hence, the transition phase is not linguistically empty but already contains a lot of linguistic information (change of handshape, direction of movement, etc.) that can be used to identify the upcoming sign (Emmorey and Corina, 1990; Hosemann et al., 2013), and thus raises some conceptual and practical issues for empirical studies and corpus linguistics. Let us briefly discuss three problems here: (1) In sign languages, the presentation of complex stimuli sign by sign is problematic since the hands must either go back to a neutral position in the signing space or the videos are cut in the middle of the transition phase. Both options are highly artificial because the transition phase connecting two signs is missing or interrupted. Moreover, additional non-manual markers may simultaneously scope over more than one sign, which makes a presentation of complex stimuli sign by sign even more unnatural. (2) In corpus annotation, we are faced with the problem of identifying the sign on- and offset (Hanke et al., 2012). A too strict definition of on- and offset would leave us with a lot of intermediate material, the transition phase, that does not have any linguistic value. A flexible definition leaves us with the problem that on- and offset can only be identified in context and may vary between examples and annotators. In both cases, this may falsify the results of statistical evaluations of corpus data. (3) In online studies, the identification of the sign onset directly affects the time-locked evaluation of the experimental data. However, for the data evaluation, the experimenter must decide which point in time s/he uses to identify the onset of a sign. Things are even more complex since the recognition of the onset of a sign by the subjects may vary from experiment to experiment. The recognition of an upcoming event (i.e., a sign) can depend on information available in context, on information provided simultaneously by manual and non-manual activities and on properties of the critical sign itself. Therefore, the experimenter should handle this problem carefully and transparently. A related practical aspect is that in corpora and experiments, the sign onset and the trigger (i.e., the time-locked position) have to be identified manually in the videos. This means that sign language competent annotators have to determine the relevant points in time in each video frame by frame, which is a highly time-consuming task (for a discussion of trigger identification in ERP experiments, cf. Hosemann et al., 2013, 2018).

Video stimuli pose yet another challenge for another kind of experimental online studies, namely eye tracking studies. In eye-tracking experiments on sign languages, typical measures such as fixation and saccades are more difficult to define and to relate to the linguistic input than in typical eye-tracking experiments on spoken languages that present the input in written form. This might be one reason why up to now only a few eye tracking studies on sign languages have been conducted. These studies focus either on eye gaze of signers during production (Thompson et al., 2006, 2009; Hosemann, 2011) or on the question whether the addressee typically focuses on the face of the signer (Muir and Richardson, 2005; Emmorey et al., 2009). Very few studies conducted a visual world experiment where the visually presented items are not linguistic objects (e.g., individual signs or complex utterances) but pictures somehow related to the linguistic input (Thompson et al., 2013; Lieberman et al., 2015, 2017). Hence, on the one hand, the lack of a writing system prevents the linguistic study of eye movements during processing the written form of a language. This means that standard methods, which are well established in psycholinguistic research on the written form of spoken languages, cannot be applied to sign languages.^6^ On the other, the presentation of visual stimuli (i.e., videos of naturally signed stimuli) makes the definition of areas of interests over many different stimuli and the linguistic evaluation of additional eye movements related to the linguistic input (e.g., in a visual world paradigm) more difficult. Hence, specific properties of the visual-gestural modality complicate the applicability of a standard online technique of experimental linguistic research.

Let us finally turn to the impact of the three modality-specific properties mentioned in the introduction: simultaneity, space, and gesture. All three properties require smart theoretical decisions and they cause extra effort in the transcription and annotation of linguistic examples collected in a corpus or in a production study (Frishberg et al., 2012; Orfanidou et al., 2015). On the one hand, the form and function of simultaneously used articulators need to be annotated on different tiers. Since these articulators express grammatical properties at different linguistic levels (prosody, morphology, syntax, semantics, and pragmatics) and interact in non-trivial ways, even simple examples require complex annotations (for a discussion of the annotation of action role shift, cf. e.g., Cormier et al., 2015). Given the fact that automatic segmentation and annotation are not available for sign language data yet, it is obvious that the complex annotation sequences of sign language data is extremely time consuming. A similar problem is the mapping of three-dimensional properties of the signing space onto a two-dimensional linguistic annotation schema. This does not only concern phonological properties of lexical signs but also grammatical features realized in the signing space. On the other hand, manual and non-manual gestures and signs are not always easy to distinguish (Goldin-Meadow and Brentari, 2017). This leads to the modality-specific problem to integrate gestures or gesture-like elements at various levels into the linguistic annotation. This problem presupposes, however, a clear theoretical definition of gesture and sign as well as the interaction of gestures and signs.

The three modality-specific properties also raise interesting questions for experimental studies and make cross-modal comparisons between spoken and sign languages difficult. Let us consider simultaneity and space first. Since sign languages use spatial and simultaneous markers to realize grammatical features, the creation of controlled stimuli is not always easy. Spatial grammatical features such as R(eferential)-loci can, for instance, be marked manually (movement and orientation of agreement verbs) or non-manually (body lean, head movement, or eye gaze). Experimental studies on the use of R-loci, may, for example, require the control of simultaneous non-manual identification of R-loci in the stimuli to investigate the manual grammatical properties of pronouns or agreement verbs. Hence, the experimenter may decide to neutralize the non-manual markers in the examples. This may, however, result in quite unnatural stimuli and thus affect the results of the experiment (cf. Hosemann et al., 2018; Wienholz et al., 2018). The same holds true for other non-manuals such as mouthing or facial expressions. A related problem is that spatial features cannot be neutralized completely since any sign is produced in space. Therefore, even the production of a simple sign may affect spatial interpretations. By contrast, if we only use natural stimuli in experimental studies, we may not be able to control the stimuli to the extent necessary for a valid and reliable evaluation of the data. A similar problem can arise from the use of iconic signs and gestural elements in sign language, which may affect grammaticality judgments and trigger different paths of processing.

## Summary

In this article, we have shown that sign language linguists are faced with a number of challenges that are either related to socio-linguistic aspects of the signing community (the data source problem) or to specific linguistic aspects of the visual-gestural modality and to methodological problems of sign language data collection, annotation, and stimuli creation (modality and data collection). In addition, we have argued that while some of these challenges also concern linguistic studies of spoken languages (particularly, of spoken varieties of small communities with no written tradition, such as in the so-called Italian dialects), other challenges are more modality-specific. Therefore, studies on sign languages are typically much more time-consuming than comparable investigations of spoken languages, especially of well-established and well-documented spoken languages. However, facing these challenges is worth the effort, since the expertise gained in empirical and experimental studies of sign languages and sign language documentation (reference grammars and corpora), while germane in several respects to empirical research in small spoken language communities is in other respects pioneering work and will pave the way for future multimodal investigations of spoken languages including co-speech gestures and facial expressions.

## Author Contributions

Both authors JQ and MS have contributed equally to this article.

### Conflict of Interest Statement

The authors declare that the research was conducted in the absence of any commercial or financial relationships that could be construed as a potential conflict of interest.

## Footnotes

^1^This view is not fully shared by some sign language scholars working in cognitive linguistics, who put the emphasis on the differences derived from the visual-gestural modality (cf. Liddell, 2003, a.o.).

^2^This amounts to Labov’s Consensus Principle: “If there is no reason to think otherwise, assume that the judgments of any native speaker are characteristic of all speakers of the language” (Labov, 1996).

^3^For an overview, see https://www.sign-lang.uni-hamburg.de/dgs-korpus/index.php/sl-corpora.html.

^4^Two groups deserve closer attention, namely Deaf children and individuals with impairments in sign languages, which usually neither receive assessment nor intervention (for more information on ethics issues, see also Baker (2012) and the ethics statement of the Sign Language Linguistics Society: http://slls.eu/slls-ethics-statement/)

^5^One reviewer mentioned that even in spoken languages, onset time is not always strictly accurate. While this is definitely true, the determination of the sign onset poses quantitatively and qualitatively different (and more serious) problems in sign languages as compared to spoken languages. In addition, the development of automatic sign processing and parsing is still in the very early stage, which means that research on sign languages, unlike research on spoken languages, will require a lot of manual annotation even in the near future (cf. Sáfár and Glauert, 2012; Hanke, 2016 and the discussion below).

^6^This is, of course, also true for the processing of auditory stimuli. Note, however, that psycholinguistic studies on many (but not all) spoken languages can use two different input modalities (i.e., written and spoken modality) to investigate linguistic structure. By contrast, psycholinguistic studies on sign languages cannot draw on written stimuli. This makes a big difference for psycholinguistic investigations. The huge amount of psycholinguistic research on written language shows that written stimuli can successfully be used to get insight in the processing of spoken languages in general (although the written modality is not a simple copy of the spoken modality).
